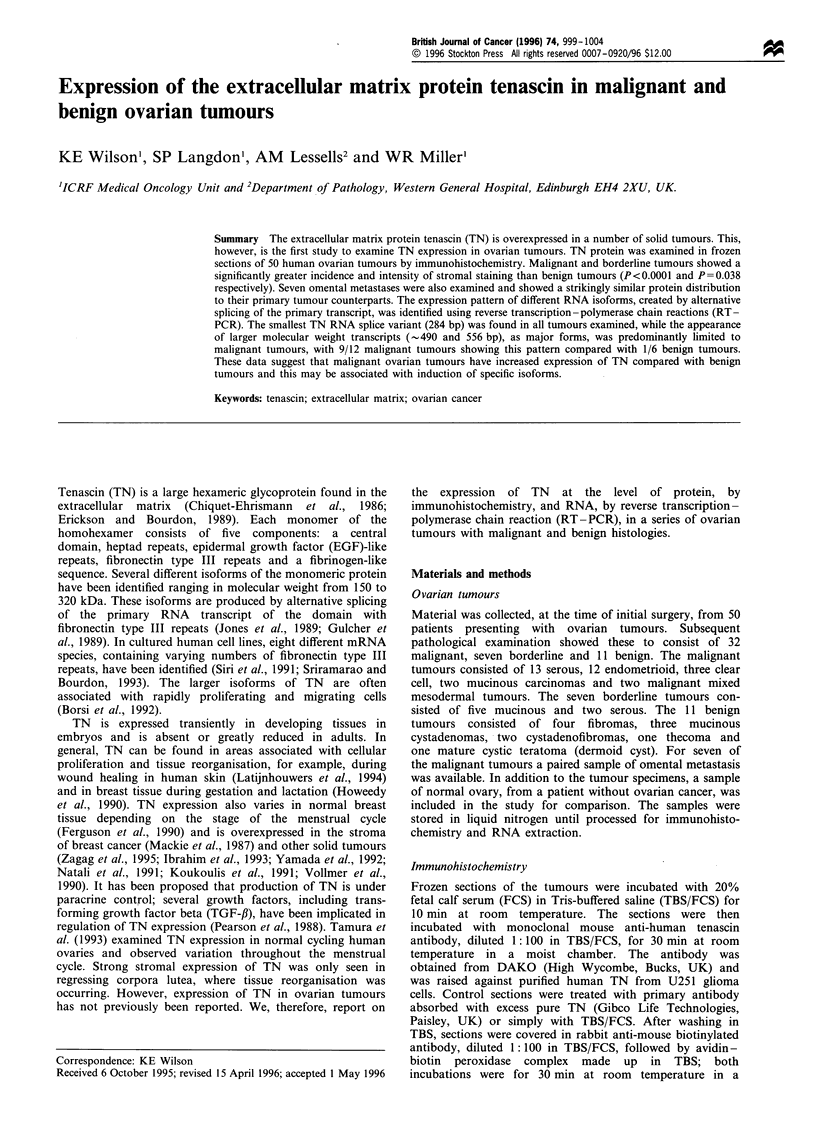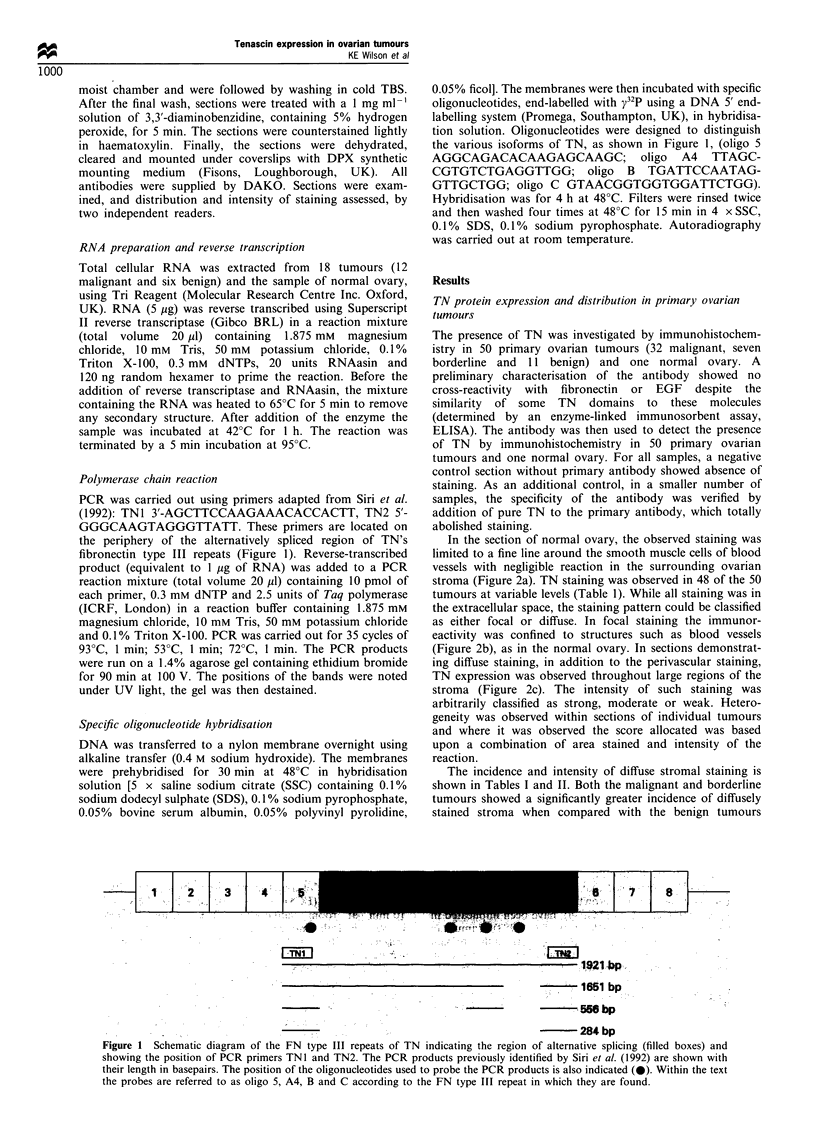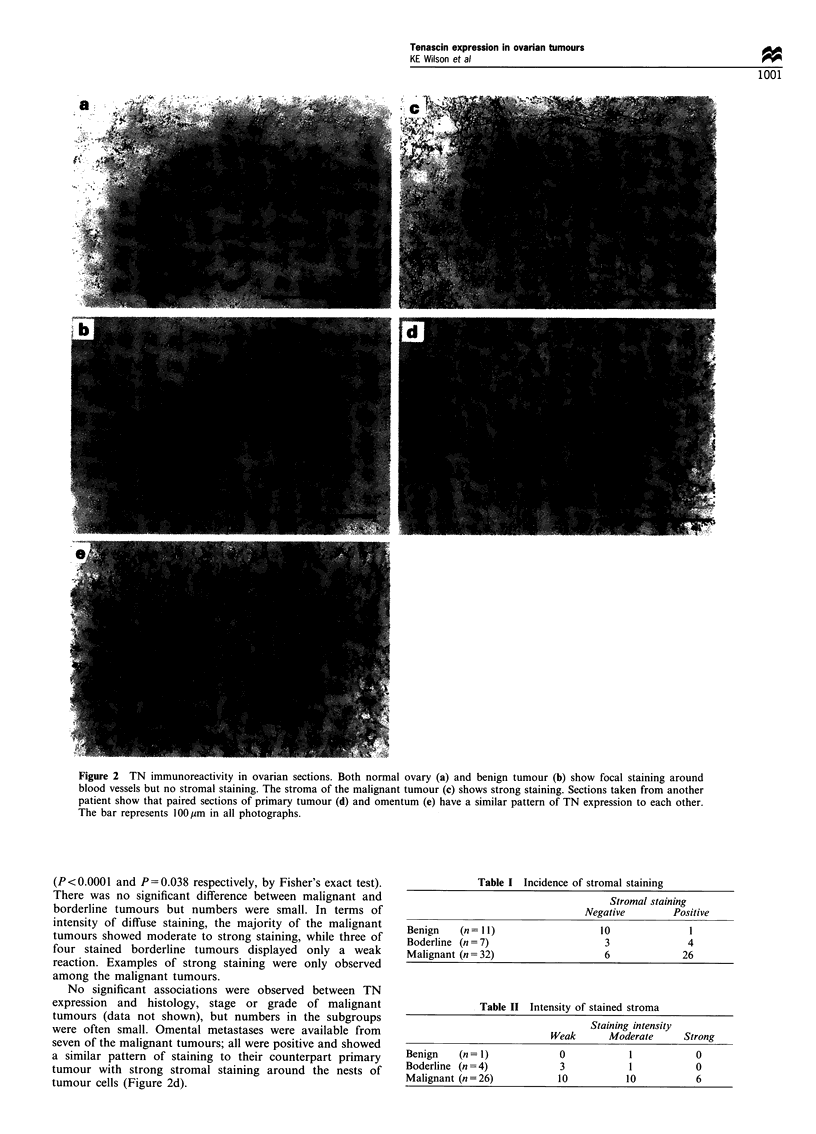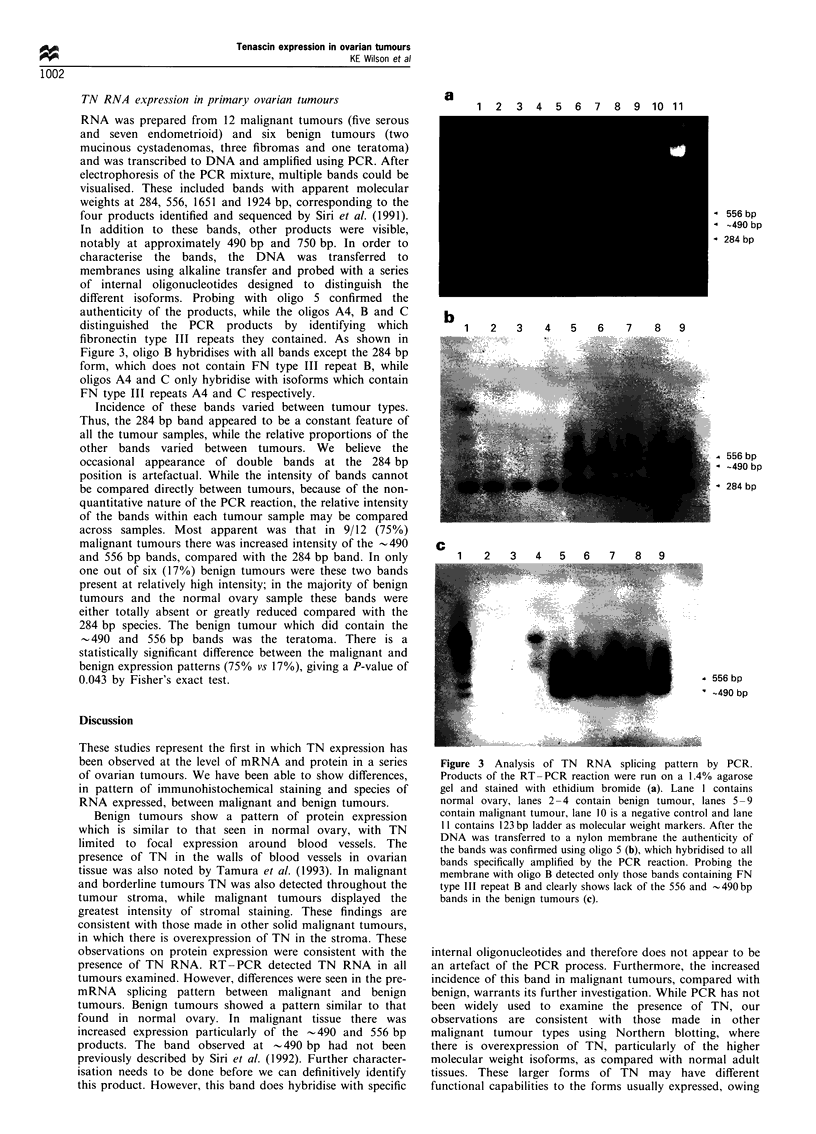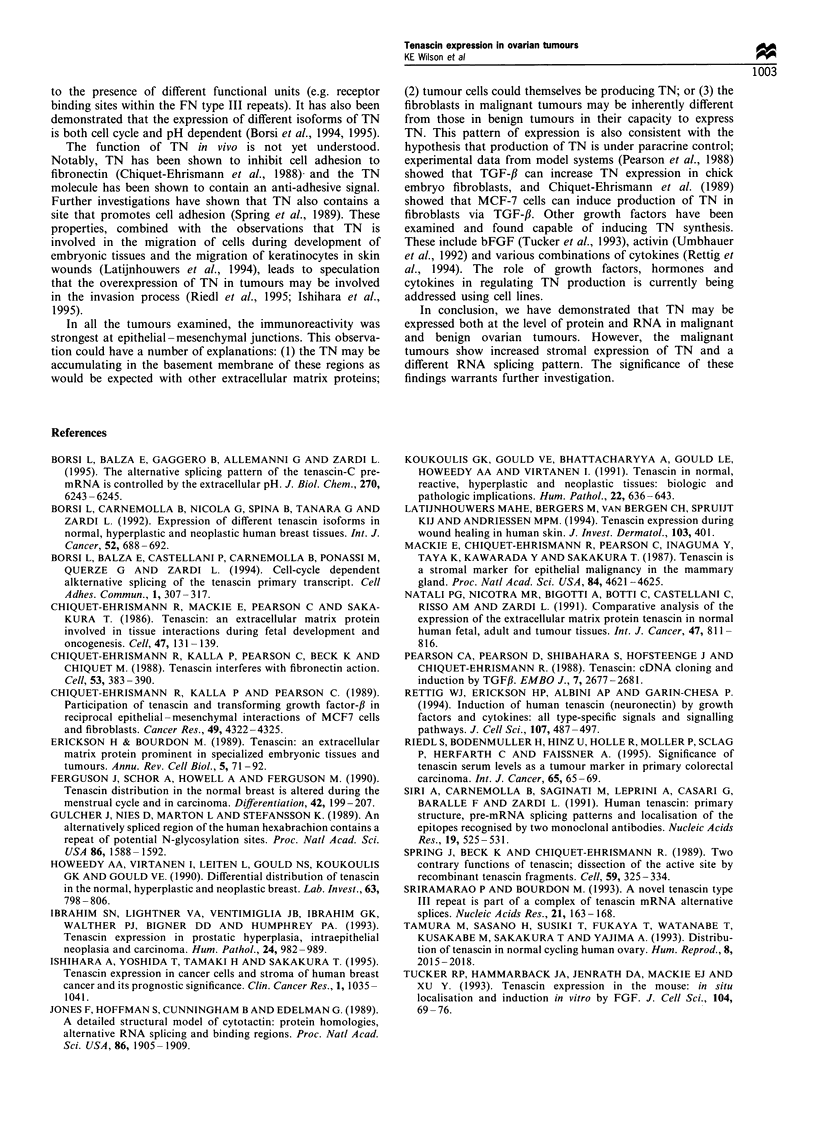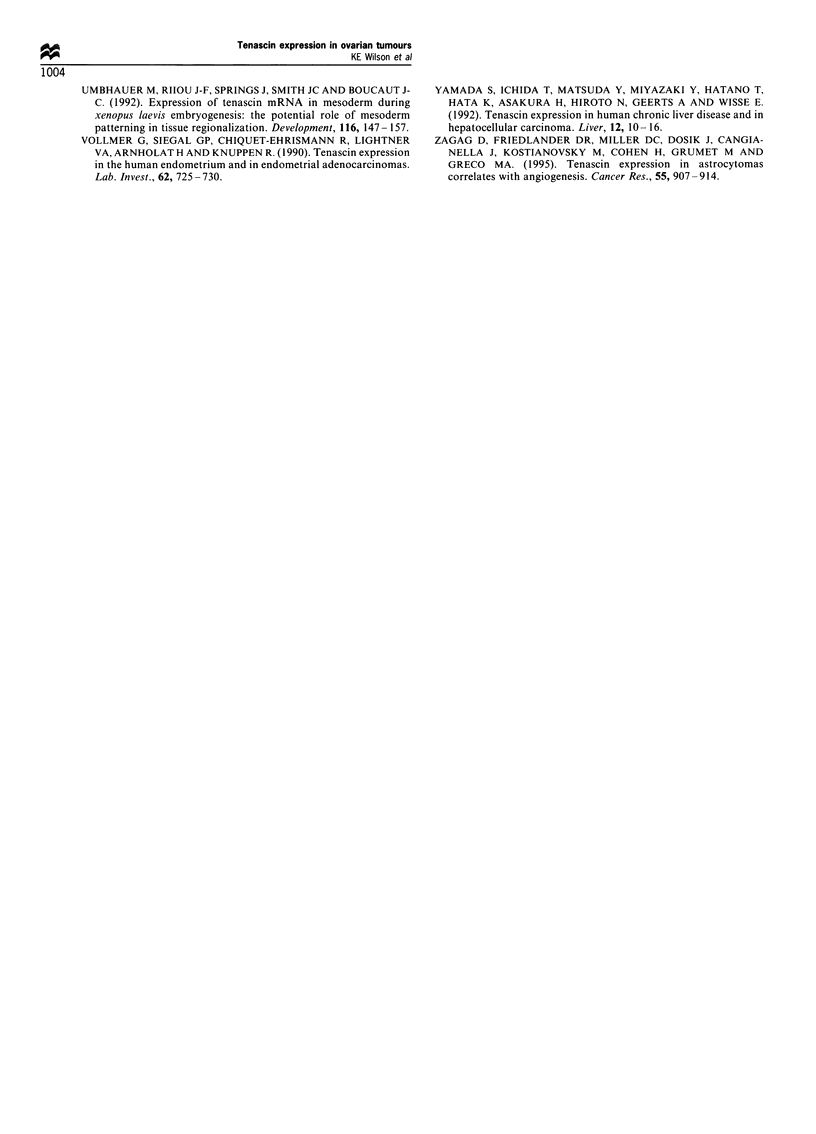# Expression of the extracellular matrix protein tenascin in malignant and benign ovarian tumours.

**DOI:** 10.1038/bjc.1996.480

**Published:** 1996-10

**Authors:** K. E. Wilson, S. P. Langdon, A. M. Lessells, W. R. Miller

**Affiliations:** ICRF Medical Oncology Unit, Western General Hospital, Edinburgh, UK.

## Abstract

**Images:**


British Journal of Cancer (1996) 74, 999-1004
© 1996 Stockton Press All rights reserved 0007-0920/96 $12.00

### Expression of the extracellular matrix protein tenascin in malignant and benign ovarian tumours

KE Wilson[1], SP Langdon[1], AM Lessells[2] and WR Miller[1]

[1]*ICRF Medical Oncology Unit and* [2]*Department of Pathology, Western General Hospital, Edinburgh EH4 2XU, UK.*

**Summary** The extracellular matrix protein tenascin (TN) is overexpressed in a number of solid tumours. This, however, is the first study to examine TN expression in ovarian tumours. TN protein was examined in frozen sections of 50 human ovarian tumours by immunohistochemistry. Malignant and borderline tumours showed a significantly greater incidence and intensity of stromal staining than benign tumours ($P<0.0001$ and $P=0.038$ respectively). Seven omental metastases were also examined and showed a strikingly similar protein distribution to their primary tumour counterparts. The expression pattern of different RNA isoforms, created by alternative splicing of the primary transcript, was identified using reverse transcription-polymerase chain reactions (RT-PCR). The smallest TN RNA splice variant (284 bp) was found in all tumours examined, while the appearance of larger molecular weight transcripts (~490 and 556 bp), as major forms, was predominantly limited to malignant tumours, with 9/12 malignant tumours showing this pattern compared with 1/6 benign tumours. These data suggest that malignant ovarian tumours have increased expression of TN compared with benign tumours and this may be associated with induction of specific isoforms.

**Keywords:** tenascin; extracellular matrix; ovarian cancer

Tenascin (TN) is a large hexameric glycoprotein found in the extracellular matrix (Chiquet-Ehrismann *et al.*, 1986; Erickson and Bourdon, 1989). Each monomer of the homohexamer consists of five components: a central domain, heptad repeats, epidermal growth factor (EGF)-like repeats, fibronectin type III repeats and a fibrinogen-like sequence. Several different isoforms of the monomeric protein have been identified ranging in molecular weight from 150 to 320 kDa. These isoforms are produced by alternative splicing of the primary RNA transcript of the domain with fibronectin type III repeats (Jones *et al.*, 1989; Gulcher *et al.*, 1989). In cultured human cell lines, eight different mRNA species, containing varying numbers of fibronectin type III repeats, have been identified (Siri *et al.*, 1991; Sriramarao and Bourdon, 1993). The larger isoforms of TN are often associated with rapidly proliferating and migrating cells (Borsi *et al.*, 1992).

TN is expressed transiently in developing tissues in embryos and is absent or greatly reduced in adults. In general, TN can be found in areas associated with cellular proliferation and tissue reorganisation, for example, during wound healing in human skin (Latijnhouwers *et al.*, 1994) and in breast tissue during gestation and lactation (Howeedy *et al.*, 1990). TN expression also varies in normal breast tissue depending on the stage of the menstrual cycle (Ferguson *et al.*, 1990) and is overexpressed in the stroma of breast cancer (Mackie *et al.*, 1987) and other solid tumours (Zagag *et al.*, 1995; Ibrahim *et al.*, 1993; Yamada *et al.*, 1992; Natali *et al.*, 1991; Koukoulis *et al.*, 1991; Vollmer *et al.*, 1990). It has been proposed that production of TN is under paracrine control; several growth factors, including transforming growth factor beta (TGF-$\beta$), have been implicated in regulation of TN expression (Pearson *et al.*, 1988). Tamura *et al.* (1993) examined TN expression in normal cycling human ovaries and observed variation throughout the menstrual cycle. Strong stromal expression of TN was only seen in regressing corpora lutea, where tissue reorganisation was occurring. However, expression of TN in ovarian tumours has not previously been reported. We, therefore, report on the expression of TN at the level of protein, by immunohistochemistry, and RNA, by reverse transcription-polymerase chain reaction (RT-PCR), in a series of ovarian tumours with malignant and benign histologies.

Correspondence: KE Wilson
Received 6 October 1995; revised 15 April 1996; accepted 1 May 1996

### Materials and methods

### *Ovarian tumours*

Material was collected, at the time of initial surgery, from 50 patients presenting with ovarian tumours. Subsequent pathological examination showed these to consist of 32 malignant, seven borderline and 11 benign. The malignant tumours consisted of 13 serous, 12 endometrioid, three clear cell, two mucinous carcinomas and two malignant mixed mesodermal tumours. The seven borderline tumours consisted of five mucinous and two serous. The 11 benign tumours consisted of four fibromas, three mucinous cystadenomas, two cystadenofibromas, one thecoma and one mature cystic teratoma (dermoid cyst). For seven of the malignant tumours a paired sample of omental metastasis was available. In addition to the tumour specimens, a sample of normal ovary, from a patient without ovarian cancer, was included in the study for comparison. The samples were stored in liquid nitrogen until processed for immunohistochemistry and RNA extraction.

### *Immunohistochemistry*

Frozen sections of the tumours were incubated with 20% fetal calf serum (FCS) in Tris-buffered saline (TBS/FCS) for 10 min at room temperature. The sections were then incubated with monoclonal mouse anti-human tenascin antibody, diluted 1:100 in TBS/FCS, for 30 min at room temperature in a moist chamber. The antibody was obtained from DAKO (High Wycombe, Bucks, UK) and was raised against purified human TN from U251 glioma cells. Control sections were treated with primary antibody absorbed with excess pure TN (Gibco Life Technologies, Paisley, UK) or simply with TBS/FCS. After washing in TBS, sections were covered in rabbit anti-mouse biotinylated antibody, diluted 1:100 in TBS/FCS, followed by avidin-biotin peroxidase complex made up in TBS; both incubations were for 30 min at room temperature in a moist chamber and were followed by washing in cold TBS. After the final wash, sections were treated with a 1 mg $ml^{-1}$ solution of 3,3′-diaminobenzidine, containing 5% hydrogen peroxide, for 5 min. The sections were counterstained lightly in haematoxylin. Finally, the sections were dehydrated, cleared and mounted under coverslips with DPX synthetic mounting medium (Fisons, Loughborough, UK). All antibodies were supplied by DAKO. Sections were examined, and distribution and intensity of staining assessed, by two independent readers.

*RNA preparation and reverse transcription*

Total cellular RNA was extracted from 18 tumours (12 malignant and six benign) and the sample of normal ovary, using Tri Reagent (Molecular Research Centre Inc. Oxford, UK). RNA (5 $\mu$g) was reverse transcribed using Superscript II reverse transcriptase (Gibco BRL) in a reaction mixture (total volume 20 $\mu$l) containing 1.875 mM magnesium chloride, 10 mM Tris, 50 mM potassium chloride, 0.1% Triton X-100, 0.3 mM dNTPs, 20 units RNAasin and 120 ng random hexamer to prime the reaction. Before the addition of reverse transcriptase and RNAasin, the mixture containing the RNA was heated to 65°C for 5 min to remove any secondary structure. After addition of the enzyme the sample was incubated at 42°C for 1 h. The reaction was terminated by a 5 min incubation at 95°C.

*Polymerase chain reaction*

PCR was carried out using primers adapted from Siri *et al.* (1992): TN1 3′-AGCTTCCAAGAAACACCACTT, TN2 5′-GGGCAAGTAGGGTTATT. These primers are located on the periphery of the alternatively spliced region of TN's fibronectin type III repeats (Figure 1). Reverse-transcribed product (equivalent to 1 $\mu$g of RNA) was added to a PCR reaction mixture (total volume 20 $\mu$l) containing 10 pmol of each primer, 0.3 mM dNTP and 2.5 units of *Taq* polymerase (ICRF, London) in a reaction buffer containing 1.875 mM magnesium chloride, 10 mM Tris, 50 mM potassium chloride and 0.1% Triton X-100. PCR was carried out for 35 cycles of 93°C, 1 min; 53°C, 1 min; 72°C, 1 min. The PCR products were run on a 1.4% agarose gel containing ethidium bromide for 90 min at 100 V. The positions of the bands were noted under UV light, the gel was then destained.

*Specific oligonucleotide hybridisation*

DNA was transferred to a nylon membrane overnight using alkaline transfer (0.4 M sodium hydroxide). The membranes were prehybridised for 30 min at 48°C in hybridisation solution [5 × saline sodium citrate (SSC) containing 0.1% sodium dodecyl sulphate (SDS), 0.1% sodium pyrophosphate, 0.05% bovine serum albumin, 0.05% polyvinyl pyrolidine, 0.05% ficol]. The membranes were then incubated with specific oligonucleotides, end-labelled with $\gamma^{32}P$ using a DNA 5′ end-labelling system (Promega, Southampton, UK), in hybridisation solution. Oligonucleotides were designed to distinguish the various isoforms of TN, as shown in Figure 1, (oligo 5 AGGCAGACACAAGAGCAAGC; oligo A4 TTAGC-CGTGTCTGAGGTTGG; oligo B TGATTCCAATAG-GTTGCTGG; oligo C GTAACGGTGGTGGATTCTGG). Hybridisation was for 4 h at 48°C. Filters were rinsed twice and then washed four times at 48°C for 15 min in 4 ×SSC, 0.1% SDS, 0.1% sodium pyrophosphate. Autoradiography was carried out at room temperature.

### Results

*TN protein expression and distribution in primary ovarian tumours*

The presence of TN was investigated by immunohistochemistry in 50 primary ovarian tumours (32 malignant, seven borderline and 11 benign) and one normal ovary. A preliminary characterisation of the antibody showed no cross-reactivity with fibronectin or EGF despite the similarity of some TN domains to these molecules (determined by an enzyme-linked immunosorbent assay, ELISA). The antibody was then used to detect the presence of TN by immunohistochemistry in 50 primary ovarian tumours and one normal ovary. For all samples, a negative control section without primary antibody showed absence of staining. As an additional control, in a smaller number of samples, the specificity of the antibody was verified by addition of pure TN to the primary antibody, which totally abolished staining.

In the section of normal ovary, the observed staining was limited to a fine line around the smooth muscle cells of blood vessels with negligible reaction in the surrounding ovarian stroma (Figure 2a). TN staining was observed in 48 of the 50 tumours at variable levels (Table 1). While all staining was in the extracellular space, the staining pattern could be classified as either focal or diffuse. In focal staining the immunoreactivity was confined to structures such as blood vessels (Figure 2b), as in the normal ovary. In sections demonstrating diffuse staining, in addition to the perivascular staining, TN expression was observed throughout large regions of the stroma (Figure 2c). The intensity of such staining was arbitrarily classified as strong, moderate or weak. Heterogeneity was observed within sections of individual tumours and where it was observed the score allocated was based upon a combination of area stained and intensity of the reaction.

The incidence and intensity of diffuse stromal staining is shown in Tables I and II. Both the malignant and borderline tumours showed a significantly greater incidence of diffusely stained stroma when compared with the benign tumours

**Figure 1** Schematic diagram of the FN type III repeats of TN indicating the region of alternative splicing (filled boxes) and showing the position of PCR primers TN1 and TN2. The PCR products previously identified by Siri *et al.* (1992) are shown with their length in basepairs. The position of the oligonucleotides used to probe the PCR products is also indicated (●). Within the text the probes are referred to as oligo 5, A4, B and C according to the FN type III repeat in which they are found.

**Figure 2** TN immunoreactivity in ovarian sections. Both normal ovary (**a**) and benign tumour (**b**) show focal staining around blood vessels but no stromal staining. The stroma of the malignant tumour (**c**) shows strong staining. Sections taken from another patient show that paired sections of primary tumour (**d**) and omentum (**e**) have a similar pattern of TN expression to each other. The bar represents 100 $\mu$m in all photographs.

($P<0.0001$ and $P=0.038$ respectively, by Fisher's exact test). There was no significant difference between malignant and borderline tumours but numbers were small. In terms of intensity of diffuse staining, the majority of the malignant tumours showed moderate to strong staining, while three of four stained borderline tumours displayed only a weak reaction. Examples of strong staining were only observed among the malignant tumours.

No significant associations were observed between TN expression and histology, stage or grade of malignant tumours (data not shown), but numbers in the subgroups were often small. Omental metastases were available from seven of the malignant tumours; all were positive and showed a similar pattern of staining to their counterpart primary tumour with strong stromal staining around the nests of tumour cells (Figure 2d).

**Table I** Incidence of stromal staining

| | *Stromal staining* | |
|---|---|---|
| | *Negative* | *Positive* |
| Benign ($n=11$) | 10 | 1 |
| Boderline ($n=7$) | 3 | 4 |
| Malignant ($n=32$) | 6 | 26 |

**Table II** Intensity of stained stroma

| | *Staining intensity* | | |
|---|---|---|---|
| | *Weak* | *Moderate* | *Strong* |
| Benign ($n=1$) | 0 | 1 | 0 |
| Boderline ($n=4$) | 3 | 1 | 0 |
| Malignant ($n=26$) | 10 | 10 | 6 |

*TN RNA expression in primary ovarian tumours*

RNA was prepared from 12 malignant tumours (five serous and seven endometrioid) and six benign tumours (two mucinous cystadenomas, three fibromas and one teratoma) and was transcribed to DNA and amplified using PCR. After electrophoresis of the PCR mixture, multiple bands could be visualised. These included bands with apparent molecular weights at 284, 556, 1651 and 1924 bp, corresponding to the four products identified and sequenced by Siri *et al.* (1991). In addition to these bands, other products were visible, notably at approximately 490 bp and 750 bp. In order to characterise the bands, the DNA was transferred to membranes using alkaline transfer and probed with a series of internal oligonucleotides designed to distinguish the different isoforms. Probing with oligo 5 confirmed the authenticity of the products, while the oligos A4, B and C distinguished the PCR products by identifying which fibronectin type III repeats they contained. As shown in Figure 3, oligo B hybridises with all bands except the 284 bp form, which does not contain FN type III repeat B, while oligos A4 and C only hybridise with isoforms which contain FN type III repeats A4 and C respectively.

Incidence of these bands varied between tumour types. Thus, the 284 bp band appeared to be a constant feature of all the tumour samples, while the relative proportions of the other bands varied between tumours. We believe the occasional appearance of double bands at the 284 bp position is artefactual. While the intensity of bands cannot be compared directly between tumours, because of the non-quantitative nature of the PCR reaction, the relative intensity of the bands within each tumour sample may be compared across samples. Most apparent was that in 9/12 (75%) malignant tumours there was increased intensity of the ~490 and 556 bp bands, compared with the 284 bp band. In only one out of six (17%) benign tumours were these two bands present at relatively high intensity; in the majority of benign tumours and the normal ovary sample these bands were either totally absent or greatly reduced compared with the 284 bp species. The benign tumour which did contain the ~490 and 556 bp bands was the teratoma. There is a statistically significant difference between the malignant and benign expression patterns (75% *vs* 17%), giving a *P*-value of 0.043 by Fisher's exact test.

### Discussion

These studies represent the first in which TN expression has been observed at the level of mRNA and protein in a series of ovarian tumours. We have been able to show differences, in pattern of immunohistochemical staining and species of RNA expressed, between malignant and benign tumours.

Benign tumours show a pattern of protein expression which is similar to that seen in normal ovary, with TN limited to focal expression around blood vessels. The presence of TN in the walls of blood vessels in ovarian tissue was also noted by Tamura *et al.* (1993). In malignant and borderline tumours TN was also detected throughout the tumour stroma, while malignant tumours displayed the greatest intensity of stromal staining. These findings are consistent with those made in other solid malignant tumours, in which there is overexpression of TN in the stroma. These observations on protein expression were consistent with the presence of TN RNA. RT-PCR detected TN RNA in all tumours examined. However, differences were seen in the pre-mRNA splicing pattern between malignant and benign tumours. Benign tumours showed a pattern similar to that found in normal ovary. In malignant tissue there was increased expression particularly of the ~490 and 556 bp products. The band observed at ~490 bp had not been previously described by Siri *et al.* (1992). Further characterisation needs to be done before we can definitively identify this product. However, this band does hybridise with specific internal oligonucleotides and therefore does not appear to be an artefact of the PCR process. Furthermore, the increased incidence of this band in malignant tumours, compared with benign, warrants its further investigation. While PCR has not been widely used to examine the presence of TN, our observations are consistent with those made in other malignant tumour types using Northern blotting, where there is overexpression of TN, particularly of the higher molecular weight isoforms, as compared with normal adult tissues. These larger forms of TN may have different functional capabilities to the forms usually expressed, owing

**Figure 3** Analysis of TN RNA splicing pattern by PCR. Products of the RT-PCR reaction were run on a 1.4% agarose gel and stained with ethidium bromide (**a**). Lane 1 contains normal ovary, lanes 2-4 contain benign tumour, lanes 5-9 contain malignant tumour, lane 10 is a negative control and lane 11 contains 123 bp ladder as molecular weight markers. After the DNA was transferred to a nylon membrane the authenticity of the bands was confirmed using oligo 5 (**b**), which hybridised to all bands specifically amplified by the PCR reaction. Probing the membrane with oligo B detected only those bands containing FN type III repeat B and clearly shows lack of the 556 and ~490 bp bands in the benign tumours (**c**).

to the presence of different functional units (e.g. receptor binding sites within the FN type III repeats). It has also been demonstrated that the expression of different isoforms of TN is both cell cycle and pH dependent (Borsi *et al.*, 1994, 1995).

The function of TN *in vivo* is not yet understood. Notably, TN has been shown to inhibit cell adhesion to fibronectin (Chiquet-Ehrismann *et al.*, 1988) and the TN molecule has been shown to contain an anti-adhesive signal. Further investigations have shown that TN also contains a site that promotes cell adhesion (Spring *et al.*, 1989). These properties, combined with the observations that TN is involved in the migration of cells during development of embryonic tissues and the migration of keratinocytes in skin wounds (Latijnhouwers *et al.*, 1994), leads to speculation that the overexpression of TN in tumours may be involved in the invasion process (Riedl *et al.*, 1995; Ishihara *et al.*, 1995).

In all the tumours examined, the immunoreactivity was strongest at epithelial–mesenchymal junctions. This observation could have a number of explanations: (1) the TN may be accumulating in the basement membrane of these regions as would be expected with other extracellular matrix proteins; (2) tumour cells could themselves be producing TN; or (3) the fibroblasts in malignant tumours may be inherently different from those in benign tumours in their capacity to express TN. This pattern of expression is also consistent with the hypothesis that production of TN is under paracrine control; experimental data from model systems (Pearson *et al.*, 1988) showed that TGF-$\beta$ can increase TN expression in chick embryo fibroblasts, and Chiquet-Ehrismann *et al.* (1989) showed that MCF-7 cells can induce production of TN in fibroblasts via TGF-$\beta$. Other growth factors have been examined and found capable of inducing TN synthesis. These include bFGF (Tucker *et al.*, 1993), activin (Umbhauer *et al.*, 1992) and various combinations of cytokines (Rettig *et al.*, 1994). The role of growth factors, hormones and cytokines in regulating TN production is currently being addressed using cell lines.

In conclusion, we have demonstrated that TN may be expressed both at the level of protein and RNA in malignant and benign ovarian tumours. However, the malignant tumours show increased stromal expression of TN and a different RNA splicing pattern. The significance of these findings warrants further investigation.

### References

BORSI L, BALZA E, GAGGERO B, ALLEMANNI G AND ZARDI L. (1995). The alternative splicing pattern of the tenascin-C pre-mRNA is controlled by the extracellular pH. *J. Biol. Chem.*, **270**, 6243–6245.

BORSI L, CARNEMOLLA B, NICOLA G, SPINA B, TANARA G AND ZARDI L. (1992). Expression of different tenascin isoforms in normal, hyperplastic and neoplastic human breast tissues. *Int. J. Cancer*, **52**, 688–692.

BORSI L, BALZA E, CASTELLANI P, CARNEMOLLA B, PONASSI M, QUERZE G AND ZARDI L. (1994). Cell-cycle dependent alkternative splicing of the tenascin primary transcript. *Cell Adhes. Commun.*, **1**, 307–317.

CHIQUET-EHRISMANN R, MACKIE E, PEARSON C AND SAKAKURA T. (1986). Tenascin: an extracellular matrix protein involved in tissue interactions during fetal development and oncogenesis. *Cell*, **47**, 131–139.

CHIQUET-EHRISMANN R, KALLA P, PEARSON C, BECK K AND CHIQUET M. (1988). Tenascin interferes with fibronectin action. *Cell*, **53**, 383–390.

CHIQUET-EHRISMANN R, KALLA P AND PEARSON C. (1989). Participation of tenascin and transforming growth factor-$\beta$ in reciprocal epithelial–mesenchymal interactions of MCF7 cells and fibroblasts. *Cancer Res.*, **49**, 4322–4325.

ERICKSON H & BOURDON M. (1989). Tenascin: an extracellular matrix protein prominent in specialized embryonic tissues and tumours. *Annu. Rev. Cell Biol.*, **5**, 71–92.

FERGUSON J, SCHOR A, HOWELL A AND FERGUSON M. (1990). Tenascin distribution in the normal breast is altered during the menstrual cycle and in carcinoma. *Differentiation*, **42**, 199–207.

GULCHER J, NIES D, MARTON L AND STEFANSSON K. (1989). An alternatively spliced region of the human hexabrachion contains a repeat of potential N-glycosylation sites. *Proc. Natl Acad. Sci. USA* **86**, 1588–1592.

HOWEEDY AA, VIRTANEN I, LEITEN L, GOULD NS, KOUKOULIS GK AND GOULD VE. (1990). Differential distribution of tenascin in the normal, hyperplastic and neoplastic breast. *Lab. Invest.*, **63**, 798–806.

IBRAHIM SN, LIGHTNER VA, VENTIMIGLIA JB, IBRAHIM GK, WALTHER PJ, BIGNER DD AND HUMPHREY PA. (1993). Tenascin expression in prostatic hyperplasia, intraepithelial neoplasia and carcinoma. *Hum. Pathol.*, **24**, 982–989.

ISHIHARA A, YOSHIDA T, TAMAKI H AND SAKAKURA T. (1995). Tenascin expression in cancer cells and stroma of human breast cancer and its prognostic significance. *Clin. Cancer Res.*, **1**, 1035–1041.

JONES F, HOFFMAN S, CUNNINGHAM B AND EDELMAN G. (1989). A detailed structural model of cytotactin: protein homologies, alternative RNA splicing and binding regions. *Proc. Natl Acad. Sci. USA*, **86**, 1905–1909.

KOUKOULIS GK, GOULD VE, BHATTACHARYYA A, GOULD LE, HOWEEDY AA AND VIRTANEN I. (1991). Tenascin in normal, reactive, hyperplastic and neoplastic tissues: biologic and pathologic implications. *Hum. Pathol.*, **22**, 636–643.

LATIJNHOUWERS MAHE, BERGERS M, VAN BERGEN CH, SPRUIJT KIJ AND ANDRIESSEN MPM. (1994). Tenascin expression during wound healing in human skin. *J. Invest. Dermatol.*, **103**, 401.

MACKIE E, CHIQUET-EHRISMANN R, PEARSON C, INAGUMA Y, TAYA K, KAWARADA Y AND SAKAKURA T. (1987). Tenascin is a stromal marker for epithelial malignancy in the mammary gland. *Proc. Natl Acad. Sci. USA*, **84**, 4621–4625.

NATALI PG, NICOTRA MR, BIGOTTI A, BOTTI C, CASTELLANI C, RISSO AM AND ZARDI L. (1991). Comparative analysis of the expression of the extracellular matrix protein tenascin in normal human fetal, adult and tumour tissues. *Int. J. Cancer*, **47**, 811–816.

PEARSON CA, PEARSON D, SHIBAHARA S, HOFSTEENGE J AND CHIQUET-EHRISMANN R. (1988). Tenascin: cDNA cloning and induction by TGF$\beta$. *EMBO J.*, **7**, 2677–2681.

RETTIG WJ, ERICKSON HP, ALBINI AP AND GARIN-CHESA P. (1994). Induction of human tenascin (neuronectin) by growth factors and cytokines: all type-specific signals and signalling pathways. *J. Cell Sci.*, **107**, 487–497.

RIEDL S, BODENMULLER H, HINZ U, HOLLE R, MOLLER P, SCLAG P, HERFARTH C AND FAISSNER A. (1995). Significance of tenascin serum levels as a tumour marker in primary colorectal carcinoma. *Int. J. Cancer*, **65**, 65–69.

SIRI A, CARNEMOLLA B, SAGINATI M, LEPRINI A, CASARI G, BARALLE F AND ZARDI L. (1991). Human tenascin: primary structure, pre-mRNA splicing patterns and localisation of the epitopes recognised by two monoclonal antibodies. *Nucleic Acids Res.*, **19**, 525–531.

SPRING J, BECK K AND CHIQUET-EHRISMANN R. (1989). Two contrary functions of tenascin; dissection of the active site by recombinant tenascin fragments. *Cell*, **59**, 325–334.

SRIRAMARAO P AND BOURDON M. (1993). A novel tenascin type III repeat is part of a complex of tenascin mRNA alternative splices. *Nucleic Acids Res.*, **21**, 163–168.

TAMURA M, SASANO H, SUSIKI T, FUKAYA T, WATANABE T, KUSAKABE M, SAKAKURA T AND YAJIMA A. (1993). Distribution of tenascin in normal cycling human ovary. *Hum. Reprod.*, **8**, 2015–2018.

TUCKER RP, HAMMARBACK JA, JENRATH DA, MACKIE EJ AND XU Y. (1993). Tenascin expression in the mouse: *in situ* localisation and induction *in vitro* by FGF. *J. Cell Sci.*, **104**, 69–76.

UMBHAUER M, RIIOU J-F, SPRINGS J, SMITH JC AND BOUCAUT J-C. (1992). Expression of tenascin mRNA in mesoderm during *xenopus laevis* embryogenesis: the potential role of mesoderm patterning in tissue regionalization. *Development*, **116,** 147–157.

VOLLMER G, SIEGAL GP, CHIQUET-EHRISMANN R, LIGHTNER VA, ARNHOLAT H AND KNUPPEN R. (1990). Tenascin expression in the human endometrium and in endometrial adenocarcinomas. *Lab. Invest.*, **62,** 725–730.

YAMADA S, ICHIDA T, MATSUDA Y, MIYAZAKI Y, HATANO T, HATA K, ASAKURA H, HIROTO N, GEERTS A AND WISSE E. (1992). Tenascin expression in human chronic liver disease and in hepatocellular carcinoma. *Liver*, **12,** 10–16.

ZAGAG D, FRIEDLANDER DR, MILLER DC, DOSIK J, CANGIANELLA J, KOSTIANOVSKY M, COHEN H, GRUMET M AND GRECO MA. (1995). Tenascin expression in astrocytomas correlates with angiogenesis. *Cancer Res.*, **55,** 907–914.